# Strategy for the Construction of SARS-CoV-2 S and N Recombinant Proteins and Their Immunogenicity Evaluation

**DOI:** 10.3390/biotech14020038

**Published:** 2025-05-23

**Authors:** Paulo Henrique Guilherme Borges, Barbara Gregio, Helena Tiemi Suzukawa, Gislaine Silva-Rodrigues, Emanuella de Castro Andreassa, Isabela Madeira de Castro, Guilherme Bartolomeu-Gonçalves, Emerson José Venancio, Phileno Pinge-Filho, Viviane Monteiro Góes, Celso Vataru Nakamura, Eliandro Reis Tavares, Tatiana de Arruda Campos Brasil de Souza, Sueli Fumie Yamada-Ogatta, Lucy Megumi Yamauchi

**Affiliations:** 1 Post-Graduate Program in Microbiology, Department of Microbiology, State University of Londrina, Londrina 86.055-900, Brazil; paulo.guilhermeph@uel.br (P.H.G.B.); barbara.g.gregio@gmail.com (B.G.); gislaine.srodrigues@uel.br (G.S.-R.); isabela.mcastro@uel.br (I.M.d.C.); 2 Laboratory of Molecular Biology of Microorganisms, Department of Microbiology, State University of Londrina, Londrina 86.055-900, Brazil; helena.tiemi.suzukawa@uel.br (H.T.S.); tavares.eliandro@uel.br (E.R.T.); ogatta@uel.br (S.F.Y.-O.); 3 Carlos Chagas Institute, Oswaldo Cruz Foundation (FIOCRUZ-PR), Curitiba 81350-010, Brazil; emllacastro01@gmail.com (E.d.C.A.); tatiana.brasil@gmail.com (T.d.A.C.B.d.S.); 4 Post-Graduate Program in Clinical and Laboratory Physiopathology, State University of Londrina, Londrina 86.055-900, Brazil; guilherme.bartolomeu@uel.br; 5 Department of Immunology, Parasitology and General Pathology, State University of Londrina, Londrina 86.055-900, Brazil; emersonj@uel.br (E.J.V.); pingefilho@uel.br (P.P.-F.); 6 Institute of Molecular Biology of Paraná, Curitiba 81350-010, Brazil; viviane@ibmp.org.br; 7 Laboratory of Technological Innovation in the Development of Pharmaceuticals and Cosmetics, State University of Maringá, Maringá 87020-900, Brazil; cvnakamura@uem.br; 8 Department of Medicine, Pontifical Catholic University of Paraná, Londrina 86067-000, Brazil

**Keywords:** SARS-CoV-2, immunoinformatics, purification of recombinant proteins, synthetic biology

## Abstract

This study reports the construction, expression, and purification of synthetic SARS-CoV-2 spike (S) and nucleoprotein (N) containing immunodominant epitopes. The pET28aS_epit construct included epitopes 287–317, 402, 507, 524–598, and 601–640, while the pET28aN_epit construct included residues 42–62, 153–172, and 355–401. Commercial sequences of both proteins were used as controls. The four constructs were expressed using the *Escherichia coli* BL21(DE3) star strain at 37 °C. The results show that the S protein constructs were insoluble, unlike the N protein constructs. Both recombinant proteins induced immune responses in mice and were recognized by antibodies present in sera from COVID-19-positive and/or SARS-CoV-2-vaccinated humans. No significant differences in immune recognition were observed between our constructs and the commercially available proteins. In conclusion, S_epit and N_epit could be promising starting points for the development of new strategies based on immunological reactions for the control of SARS-CoV-2 infections.

## 1. Introduction

The enveloped beta-coronavirus SARS-CoV-2 is the causative agent of COVID-19 and comprises a positive-sense single-stranded RNA genome [1,2,3] which encodes four structural proteins—spike glycoprotein (S), nucleoprotein (N), membrane protein (M), and envelope protein (E)—as well as several non-structural proteins. The structural proteins are essential for the viral entry and survival within mammalian cells, and among them, S and N proteins have been the main targets of COVID-19 vaccine development [4,5]. The S protein is the main surface antigen of SARS-CoV-2 and triggers neutralizing antibody responses [6,7]. It plays a crucial role in the interplay between SARS-CoV-2 and host cells by binding to the angiotensin-converting enzyme 2-related carboxypeptidase (ACE2) receptor, resulting in viral entry into mammalian cells. The N is the most abundant among SARS-CoV-2 proteins and is associated with viral RNA packing into the ribonucleoprotein structure, as well as viral RNA replication and transcription. Additionally, the N protein participates in the suppression of the host’s innate immune system, serving as an immunogen that elicits a T-helper1 (Th1) immune response [8,9,10,11,12].

The COVID-19 pandemic underscored the critical importance of preparedness for potential future pandemics and highlighted the importance of research involving recombinant proteins in the development of immunodiagnostic tests and vaccines, mainly those based on the S and N proteins [4,5,13]. Indeed, given their safety and low incidence of adverse effects, the relevance of protein-based vaccines justifies the study of recombinant protein constructs [14,15]. In this scenario, a critical concern includes the sustained efficacy of immune responses against variants of the aforementioned virus [16]. Hence, in silico analysis of proteins from infectious agents has been used for the development of immunodiagnostic tests and vaccines, facilitating the identification of highly immunogenic and conserved epitopes that can trigger immune responses [12,16,17].

This study reports the construction, expression, and purification of two recombinant SARS-CoV-2 proteins, S and N, containing immunodominant epitopes. Thus, an in silico analysis of S and N protein epitopes was carried out to predict B lymphocyte activation sites. The nucleotide sequences coding these proteins were inserted into a His-tag expression vector in *Escherichia coli*. Moreover, the immunoreactivity of the recombinant proteins was evaluated using mice and human sera.

## 2. Materials and Methods

### 2.1. Analysis of B-Cell Epitopes, Protein Design, and Construction of Expression Vectors for S and N Recombinant Proteins

The amino acid sequences used in this study were designed through structural modeling of the SARS-CoV-2 S and N proteins using the Modeller 10.6 software [18,19], followed by the identification of immunogenic epitope positions previously described by Grifoni et al. [12] and Yuan et al. [20]. The selected epitopes were aligned with the sequences of the S (YP_009724390.1) and N (YP_009724397.2) proteins, which guided the design of the constructs, optimizing epitope exposure and stability. Structural analysis resulted in the selection of three epitopes for each protein predicted as immunogenic by Grifoni et al. [12]. Moreover, an additional epitope, predicted as immunogenic by Yuan et al. [20], was added to the S recombinant protein. Fusion of the selected epitopes of the N protein was performed using a glycine loop, which enhances structural flexibility of the recombinant proteins.

The three-dimensional (3D) models of the S and N proteins, as well as the S_epit and N_epit constructs, were generated using Modeller 10.6 [19]. Structural visualization and annotation of selected epitopes were performed with Chimera 1.16 software [21]. The resulting models were used to assess the spatial distribution and surface exposure of the predicted immunogenic regions.

The nucleotide sequences of the selected epitopes of both proteins were inserted into the pET28a(+) expression vector between the *Nde*l and *Xho*l restriction sites, generating two constructs: pET28aS_epit and pET28aN_epit. Two additional vectors, pET28aS_control and pET28aN_control, containing the sequences of the commercial proteins, were also constructed to serve as controls. All the plasmids were acquired from the commercial supplier FastBio (https://www.fastbio.com.br/, accessed on 12 August 2020), with codon optimization for *E. coli* expression. After selecting the sequences, the position and distribution of the residues were observed in the 3D structure of the proteins using Chimera 1.16 software [21]. The physicochemical characterization of the recombinant proteins was carried out using the Expasy ProtParam tool (https://web.expasy.org/protparam/, accessed on 18 March 2021) [22]. The antigenicity of the epitopes was evaluated using the VaxiJen Server 2.0 online tool (http://www.ddg-pharmfac.net/vaxijen/VaxiJen/VaxiJen.html, accessed on 6 April 2025), with a cutoff value of 0.4 and the virus specified as the target agent to enhance prediction accuracy [23]. TMHMM v.2.0 (http://www.cbs.dtu.dk/services/TMHMM/, accessed on 6 April 2025) was used to predict the transmembrane topology of epitopes, providing probability graphs and classifying each residue as inside, outside, or transmembrane [24].

To assess the conservation of the selected epitopes across different SARS-CoV-2 variants, we retrieved the sequences of the Wuhan strain (NC_045512.2), as well as the Alpha (B.1.1.7), Beta (B.1.351), Delta (B.1.617.2), Gamma (P.1), and Omicron (BA.1) variants from the National Center for Biotechnology Information (https://www.ncbi.nlm.nih.gov/, accessed on 10 June 2022). These sequences underwent multiple sequence alignments using BioEdit 7.2.5 to verify the presence of conserved epitopes [25].

### 2.2. Expression of pET28aS_epit, pET28aN_epit, and Controls in E. coli and Recombinant Proteins Purification

Initially, two *E. coli* host strains and temperatures of 30 °C and 37 °C were used to evaluate the expression of the recombinant proteins. Thus, all plasmid constructs were inserted into host cells by transformation using the heat shock protocol [26]. The recombinant proteins were expressed in the *E. coli* BL21(DE3) star and *E. coli* BL21 (DE3) plysS strains carrying the plasmid constructs. Expression was induced by adding 1.0 mM isopropyl-β-d-thiogalactopyranoside (IPTG, Thermo Fisher Scientific, Carlsbad, CA, USA) to the log-phase bacterial cultures in Luria Bertani (LB) medium containing 50 µg/mL kanamycin (INLAB, Diadema, SP, Brazil) incubated at 37 °C for 1 h under agitation (150 rpm). The induced cultures were incubated further for 4 h at 30 °C or 37 °C, also under agitation. Subsequently, the cells were collected by centrifugation (10,000× *g* for 15 min at 4 °C), resuspended in lysis buffer A (20 mM sodium phosphate, 500 mM NaCl pH 7.5, and 1 µg/µL lysozyme), and lysed by sonication using two pulses of 15 s with 30 s intervals at 10% amplitude, employing a 3 mm microtip. For determination of protein solubility, the bacterial lysates were centrifuged (10,000× *g* for 15 min at 4 °C) and aliquots of the lysates (total proteins) and the supernatant (soluble protein) fractions were analyzed by 13% SDS-PAGE stained with Coomassie Brilliant Blue-G250 solution (INLAB, Diadema, SP, Brazil), followed by the Western blot analysis using monoclonal anti-histidine antibodies as primary antibodies (Roche, Tucson, AZ, USA), and alkaline phosphatase-conjugated anti-mouse antibody as secondary antibodies (Thermo Fisher Scientific, Rockford, IL, USA) [27].

For purification, the expression of the recombinant proteins was carried out in the best conditions [*E. coli* BL21 (DE3) star and 37 °C] in 2 L of LB medium containing kanamycin and processed as above, except that lysozyme was removed from the lysis buffer. The proteins were purified on a HisTrap HP affinity column (5 × 1 mL, GE Healthcare, Piscataway, NJ, USA) connected to the ÄKTA pure system (GE Healthcare, Freiburg, Breisgau, Germany) under non-denaturing (soluble protein) or denaturing (insoluble protein) conditions, using elution buffer B (20 mM sodium phosphate, 500 mM NaCl pH 7.5, and 2 M imidazole), according to the manufacturer’s instructions. The column flow rate was adjusted to 1 mL/min and the absorbance measured at 280 nm. A second purification step was performed with insoluble proteins via size exclusion chromatography using a Superdex 200 column (GE Healthcare, Piscataway, NJ, USA). For the purification of insoluble proteins, the protocol was similar, except that buffers A and B were supplemented with 6 M urea. All eluents were collected and analyzed by 13% SDS-PAGE. The purified proteins were dialyzed with 0.1 M phosphate-buffered saline (PBS), pH 7.5 under agitation for 16 h, and quantified using a Qubit™ 4 Fluorometer (Thermo Fisher Scientific, São Paulo, SP, Brazil).

### 2.3. Mice Immunization

Male BALB/c mice (*n* = 5, 8 to 12 weeks old) were immunized subcutaneously with purified recombinant S_epit and N_epit proteins. Briefly, the mice were immunized with 5 µg/mL of each protein emulsified with complete Freund’s adjuvants (CFA, Sigma-Aldrich/Merck, São Paulo, SP, Brazil) on day 0 and 5 µg/mL of each protein emulsified with incomplete Freund’s adjuvants (IFA, Sigma-Aldrich, St. Louis, MO, USA) on days 7 and 14. A control group received PBS alone, serving as a negative control for antibody production. On day 21, whole blood samples were collected, and sera were obtained and stored at −20 °C until use. All experiments involving mice were conducted with the approval of the Ethics Committee for the Use of Animals of the State University of Londrina (CEUA/UEL) under protocol number 052.2020. The mice were housed in standard polycarbonate cages with *ad libitum* access to food and water. Environmental conditions were maintained on a 12 h light/dark cycle at a controlled ambient temperature. Animal welfare was monitored regularly throughout the experimental period to ensure compliance with ethical guidelines.

### 2.4. Blood Samples

Whole blood samples (*n* = 45) were obtained from men and women aged 18 years or older presenting symptoms of COVID-19 or who were vaccinated. All serum samples were obtained and stored at −20 °C until use. Sample collection was approved by the Research Ethics Committee of the State University of Londrina (CEP/UEL) under document number 47784621.2.0000.5231 and approval number 4.862.243. All participants signed an informed consent form, expressing their agreement to the use of their samples and the publication of the study results.

### 2.5. ELISA

The reactivity of the recombinant proteins with the sera from the immunized animals and the human volunteers was assessed by enzyme-linked immunosorbent assay (ELISA). Briefly, Corning^®^ 96-well flat-bottom Clear Polystyrene Microplates (Merck, St. Louis, MO, USA) were sensitized for 16 h at 4 °C with 5 µg/mL of each recombinant protein, which were diluted in carbonate–bicarbonate buffer pH 9.6. The plates were washed three times with PBS containing 0.05% (*w*/*v*) Tween 20 (PBST) and incubated with a blocking solution of 5% (*w*/*v*) skimmed milk containing PBST for 2 h at room temperature. Then, 100 μL aliquots of each serum sample—mice serum diluted from 1:40 to 1:1280 (*v*/*v*) and human serum diluted 1:250 (*v*/*v*)—were incubated for 1 h at 37 °C. After washing procedures, anti-human IgG HRP or anti-mouse HRP secondary antibodies (Thermo Fisher Scientific, Rockford, IL, USA) were diluted 1:8000 (*v*/*v*) and incubated at 37 °C for 1 h. The reactions were revealed by adding o-phenylenediamine dihydrochloride (Sigma-Aldrich, Darmstadt, Germany) and H_2_O_2_, and stopped with 1 N sulfuric acid. The optical density was measured at 490 nm in a Synergy^TM^ HTX microplate reader (Agilient, Santa Clara, CA, USA).

### 2.6. Statistical Analysis

Data were evaluated by one-way analysis of variance (ANOVA), followed by Tukey’s post-test, using GraphPad Prism software, version 8.0.1. Values of *p* < 0.05 were considered statistically significant.

## 3. Results and Discussion

### 3.1. pET28aS_epit and pET28aN_epit Constructs, and In Silico Analysis of the S_ epit and N_epit Recombinant Proteins

Several studies utilized recombinant SARS-CoV-2 proteins targeting specific regions of structural proteins, such as the S1, S2, or RDB domains of the S protein [28,29,30], or the full-length N protein [28,31,32,33]. Unlike previous works, our study integrates epitope prediction within silico structural analysis prior to the synthesis of expression plasmids, ensuring a rational and robust design. Therefore, the recombinant proteins S_epit and N_epit were designed with highly immunogenic regions to optimize the induction of the immune response and improve recognition by specific antibodies.

**Figure 1:**
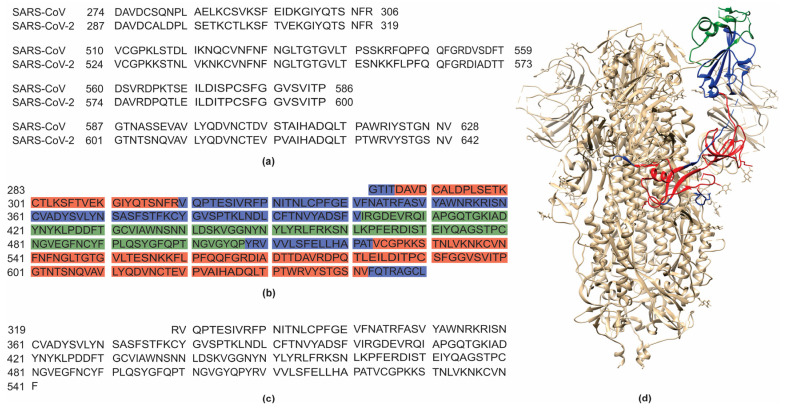
Selection and design of the SARS-CoV-2 spike protein constructs. Highly immunogenic regions were selected based on the amino acid alignment of the spike proteins of SARS-CoV and SARS-CoV-2, identified according to the amino acid position verified by Grifoni et al. [12]. The selected amino acid sequences of the SARS-CoV-2 spike protein correspond to residues 287–319, 524–600, and 601–642 (**a**). Based on these sequences, two constructs were designed: pET28aS_epit (**b**), containing residues 283–650 to encompass the selected epitopes, as well as residues 402–507, predicted as immunogenic by Yuan et al. [20]; and pET28aS_control (**c**), containing residues 319–541, corresponding to a commercially available spike protein. The structure of the SARS-CoV-2 spike protein (**d**) construct visualized in Chimera 1.16 [21], based on a predicted model generated using Modeller 10.6 [19]. Immunogenic epitopes selected for the pET28aS_epit construct are shown in red, the region 402–507 in green, and non-immunogenic but structurally relevant regions in blue.

**Figure 2:**
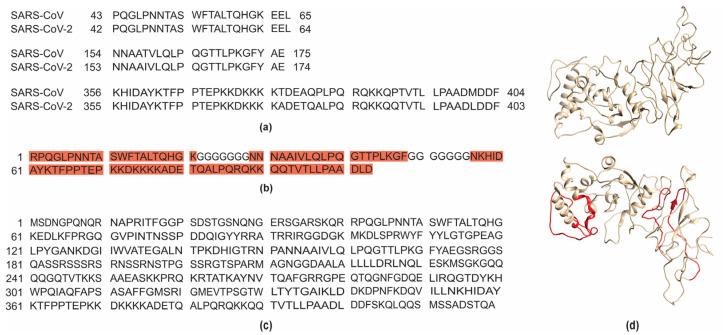
Selection and design of the SARS-CoV-2 nucleoprotein constructs. Highly immunogenic regions were selected through amino acid alignment between the SARS-CoV and SARS-CoV-2 nucleoproteins, with residues identified according to the positions reported by Grifoni et al. [12]. The selected sequences included residues 42–62, 153–172, and 355–401 (**a**). Two constructs were designed: pET28aN_epit (**b**), which include the selected epitopes linked by glycine loops; and pET28aN_control (**c**), containing the full-length amino acid sequence of the nucleoprotein. The structure of the SARS-CoV-2 nucleoprotein (**d**) construct visualized in Chimera 1.16 [21], based on a predicted model generated using Modeller 10.6 [19]. Immunogenic epitopes selected for the pET28aN_epit construct are shown in red.

The selection of the immunogenic regions within SARS-CoV-2 was based on the immunogenic residues previously identified in SARS-CoV by Grifoni et al. [12] and Yuan et al. [20] through the alignment between sequences from both viruses. This strategy was possible due to the high genetic similarity between the SARS-CoV-2 and SARS-CoV genomes [34]. Therefore, the selected regions of the S protein included residues 287–317, 524–598, and 601–640 (Figure 1a), and for the N protein, residues 42–62, 153–172, and 355–401 were selected (Figure 2a).

For the pET28aS_epit construct, a structural analysis of S protein was also carried out, which identified residue 283 as the starting amino acid to preserve the structural integrity of the beta-strand beginning at residue 287. The construct extends to residue 650, ensuring the correct folding of the C-terminal alpha helix (Figure 1b,d). In addition, the included sequence containing residues 402 to 507 was predicted as an immunogenic epitope, as supported by structural analyses in complex with neutralizing antibodies [14]. Notably, the alignment of the spike protein sequences with those of different SARS-CoV-2 variants showed a high degree of similarity within the chosen regions (Figure S1) suggesting that these sequences undergo minimal alterations and do not affect the antibody recognition in patients infected with different variants. The confirmation of conserved epitopes among SARS-CoV-2 variants strengthens the robustness of the recombinant construct design and reinforces its potential for immunological applications. As for the pET28aN_epit construct, it contains all the predicted epitope regions (residues 42–62, 153–172, and 355–401) linked by glycine loops (Figure 2b,d).

The pET28aS_control construct was designed to contain the region, spanning residues 319 to 541, along with an N-terminal domain to promote structural stabilization (Figure 1c); and the pET28aN_control construct was based on the complete theoretical sequence of the N protein (Figure 2c). The predicted 3D structures of the recombinant proteins S_epit and N_epit were also analyzed, revealing their structural alignment with the full-length SARS-CoV-2 S and N proteins, respectively (Figures S2 and S3). The visualization confirmed the surface exposure of the selected epitopes, supporting the in silico predictions of antigenicity. These findings reinforce the rational design of the constructs to preserve epitope accessibility and immune recognition potential.

In silico analysis of the physicochemical characteristics of the recombinant proteins revealed the predicted molecular weights of 40.7 kDa and 10.4 kDa, with corresponding isoelectric points of 7.52 and 9.93 for S_epit and N_epit, respectively. An estimated half-life of >10 h for S_epit and 2 min for N_epit in *E. coli* were also identified. The instability index (II) was 21.37 for S_epit (stable protein), and 51.07 for N_epit (unstable protein). Regarding antigenicity, the S_epit presented a score of 0.5851, while the N_epit scored 0.4882. Therefore, both were considered antigenic. Furthermore, membrane topology analysis confirmed that neither protein contains transmembrane domains, with all residues predicted to be exposed to the extracellular environment.

### 3.2. SARS-CoV-2 S and N Recombinant Proteins Are Expressed in Escherichia coli BL21 (DE3) Star

**Figure 3:**
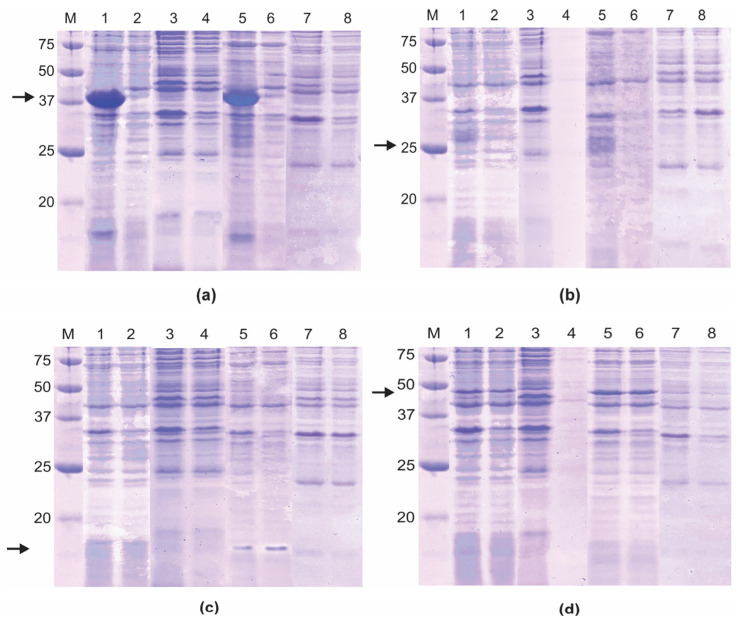
SDS-PAGE analysis of the expression of recombinant SARS-CoV-2 spike protein (**a**,**b**) and nucleoprotein (**c**,**d**) in *Escherichia coli* BL21(DE) star. Expression standardization was performed by optimizing host strains and temperature conditions. The recombinant proteins are indicated by arrows: S_epit ((**a**), 39 kDa); S_control ((**b**), 25 kDa); N_epit ((**c**), 15 kDa); and N_control ((**d**), 47 kDa). M represents the protein molecular weight marker (BioRad, 250 kDa). Total protein extracts and the supernatants, respectively, were analyzed after expression in: *E. coli* BL21 (DE3) star at 30 °C (lanes 1 and 2); *E. coli* BL21 (DE3) pLysS at 30 °C (lanes 3 and 4); *E. coli* BL21 (DE3) star at 37 °C (lanes 5 and 6); and *E. coli* BL21 (DE3) pLysS at 37 °C (lanes 7 and 8).

The expression of all plasmid constructs of S and N proteins was analyzed in two strains of *E. coli* and at two different temperatures. The results show that all S and N recombinant proteins were expressed in the *E. coli* BL21 (DE3) star strain (Figure 3). For S_epit, a protein band around 39 kDa was observed on lysates from bacteria incubated at 30 and 37 °C (Figure 3a, lanes 1 and 5, respectively), whereas the S_control protein (around 25 kDa) was expressed at 37 °C (Figure 3b, lane 5). The N_epit protein, a protein around 15 kDa, was expressed at 37 °C (Figure 3c, lanes 5 and 6), and, conversely, the N_control protein (around 47 kDa) was expressed at 30 and 37 °C (Figure 3d, lanes 1 and 2, as well as 5 and 6, respectively). Given these results, *E. coli* BL21 (DE3) star and 37 °C were selected for further analyses.

**Figure 4:**
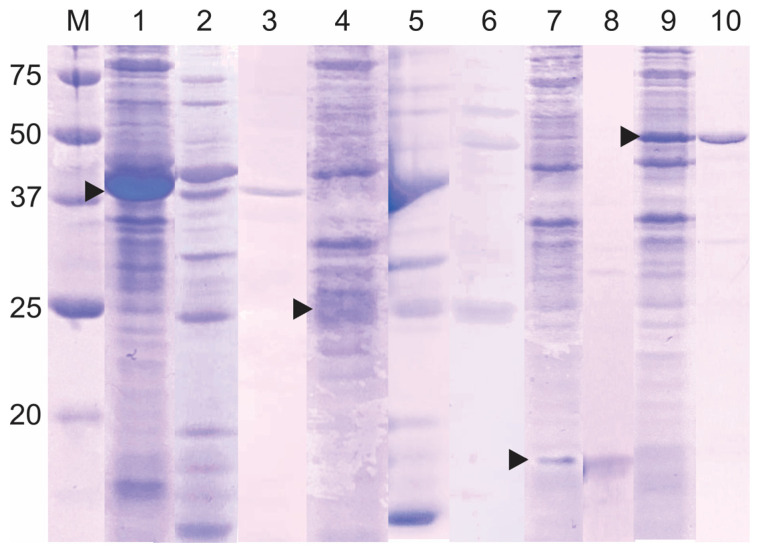
SDS-PAGE analysis showing the different stages of the recombinant SARS-CoV-2 spike proteins (lanes 1–6) and nucleoproteins (lanes 7–10) purification process. The affinity chromatography was first utilized for the purification of all proteins (lanes 2, 5, 8, and 10). Size exclusion chromatography was performed exclusively for the spike proteins (lanes 3 and 5). M represents the molecular weight marker (BioRad, 250 kDa). Lanes 1, 4, 7, and 9 show the total protein extract of *E. coli* BL21 (DE3) star at 37 °C harboring the S_epit protein (39 kDa), S_control protein (25 kDa), N_epit protein (15 kDa), and N_control protein (47 kDa) constructs, respectively. The arrowheads indicate the recombinant proteins.

Next, the solubility of the S and N recombinant proteins was analyzed directly by SDS–PAGE (Figure 4), and S_epit and S_control proteins were expressed as insoluble forms, while the N_epit and N_control proteins were expressed as soluble forms. The expression/solubility of these recombinant proteins was confirmed by Western blot analysis using monoclonal anti-histidine tag antibodies, which detected the S proteins exclusively in the total protein extract. In contrast, the N proteins were detected in both the total and soluble fractions (Figure S4). Different expression plasmid constructs for S and N proteins of SARS-CoV-2 were successfully expressed in the *E. coli* BL21 (DE3) strain at 37 °C [35,36,37], showing similar results to ours regarding protein solubility. For instance, the receptor-binding domains of S protein (amino acid 319–541, NCBI accession: NC_045512) were expressed as inclusion bodies, which were then purified under a denaturing condition [36]. On the other hand, Djukic et al. [37] reported that N protein fragment, spanning the amino acid residues 58 to 419 (UniProt ID, ID P0DTC9), was successfully expressed in *E. coli* BL21 (DE3) in a soluble form at 37 °C.

Given the simplicity and cost-effectiveness, *E. coli* remains the bacterial host of choice for the expression of recombinant proteins. However, a significant challenge in using this bacterium is the formation of proteins in insoluble aggregates [38], as occurred with the recombinant S proteins in this study. Several factors, such as amino acid composition and the expression of proteins on a large scale [39,40], can influence solubility and, consequently, interfere with purification efficiency [41,42,43,44]. A limitation of our study is that an in-depth analysis of the best conditions for expressing recombinant proteins in their soluble forms has not been carried out. Despite this limitation, all the recombinant proteins were successfully purified using a few purification steps.

Initially, the recombinant proteins were purified using the HisTrap HP affinity column, and the elution of the S_epit and S_control proteins required 15% (0.3 M) and 13.9% (0.278 M) imidazole, respectively; whereas 27% (0.54 M) and 27.5% (0.55 M) imidazole were used to elute the N_epit and N_control proteins, respectively. Purification of all proteins, in this system, was monitored by SDS-PAGE. As shown in Figure 4, several bacterial proteins were eluted together with the recombinant S proteins, requiring a second purification step, using size exclusion chromatography. In contrast, N recombinant proteins were visualized as a single protein band. In larger-scale expression systems (2 L), the yield of the N_epit protein was 4860 mg/L, while for the N_control, S_epit, and S_control proteins, it was 114 mg/L, 102 mg/L, and 154 mg/L, respectively. It is interesting to note that although the S_epit protein appears to be highly expressed, the amount of purified protein obtained was lower than that of the N_epit protein, which remained soluble throughout the process (Figure 3a). These findings suggest that the limitation in S_epit recovery is not due to low expression levels, but rather to its aggregation into inclusion bodies.

Fewer purification steps and high yields of purified protein offer advantages for scale-up processes. In this sense, several studies have shown that affinity chromatography is an efficient method for isolating recombinant proteins from SARS-CoV-2 [13,43,45,46]. Furthermore, size exclusion chromatography is a filtration-based technique that does not require broad knowledge of molecule properties to be effectively applied [47].

### 3.3. S_epit and N_epit Induce Humoral Immune Response in Mice and Are Recognized by Sera from COVID-19-Positive and/or Vaccinated Humans

**Figure 5:**
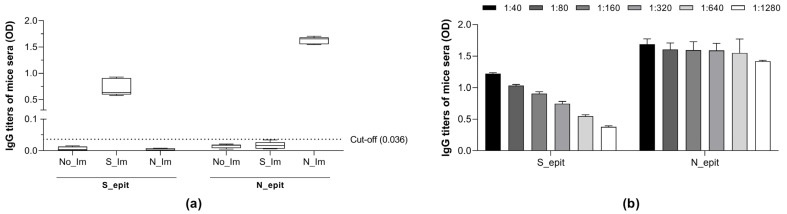
Immunogenicity of S_epit and N_epit proteins in BALB/c mice. Subcutaneous administration with both recombinant proteins was carried out, and after 21 days, post-immunization sera (1:160) were analyzed by indirect ELISA. (**a**) Indirect ELISA; (**b**) S_epit and N_epit antibodies titles. The plates were sensitized with 5 µg/mL of each recombinant proteins. No_Im: non-immunized, S_Im: S_epit-immunized, and N_Im: N_epit-immunizedmice. OD, optical density.

The humoral immune response induced by subcutaneous administration of S_epit and N_epit proteins to BALB/c mice was assessed by ELISA. Sera from non-immunized mice did not recognize the recombinant proteins, while the sera of S_epit- and N_epit-immunized mice specifically recognized their specific antigens (Figure 5a). Interestingly, the immunization of mice with the N_epit induced a higher serum titer than the S_epit protein (Figure 5b). This is consistent with previous studies demonstrating that the N protein elicits a strong immune response and is the main target for antibody recognition in SARS-CoV-2 infections [48,49,50,51,52].

**Figure 6:**
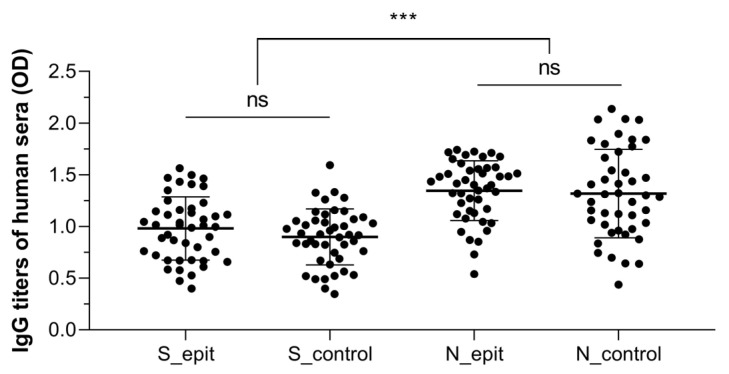
Recognition of recombinant SARS-CoV-2 spike proteins and nucleoproteins by human antibodies. Blood samples were collected from infected and/or vaccinated individuals and the sera (1:250) were analyzed by indirect ELISA. The plates were sensitized with 5 µg/mL of each recombinant proteins. *** *p* ≤ 0.001; OD, optical density; and ns, not statistically significant.

The binding capability of the S_epit and N_epit proteins to human antibodies was also evaluated. Thus, ELISA was carried out using sera from COVID-19-positive and/or vaccinated individuals, diluted 1:250. No significant differences were observed in antibody recognition between the S_epit and N_epit proteins and their respective control proteins. The average optical density values were 0.98 for S_epit, 0.90 for S_control, 1.35 for N_epit, and 1.32 for N_control (Figure 6). These data indicate that our protein constructs were sufficient to elicit an immune response and could be promising targets for antibody recognition and immune response induction strategies.

Other limitations of our study include the following: (i) A single concentration (5 µg) of the recombinant proteins was utilized to immunize the mice. Actually, the results of immunization can be affected by different variables, such as the concentration of the antigen, the choice of adjuvants, the route and the timing of both inoculation and the response measurement, and the detection method [53]. (ii) We did not investigate whether the antibodies elicited in mice are indeed protective. For studies with animals and SARS-CoV-2, a challenge will be required to confirm whether S_epit and N_epit proteins elicit protective immunity. This phase of the study will be conducted once access to a higher-level biosafety animal facility is available, as required for viral challenge experiments. (iii) The difficulty is in removing endotoxins from the *E. coli* expression system, which is widely employed due to its ease of maintenance and high productivity. In fact, obtaining endotoxin-free products remains a significant challenge, as common removal methods—such as ultrafiltration and chromatography—tend to display low efficiency [54]. Recent advances, such as the development of endotoxin-free *E. coli* strains [54] and the use of lipopolysaccharide (LPS)-free Gram-negative bacterial species [55], enabled the direct production of recombinant proteins with minimal endotoxin contamination. To address this limitation in our study, future experiments may employ these alternative bacterial systems.

Despite these limitations, our findings reveal that chimeric S and N proteins harboring immunogenic epitopes were capable of eliciting a B-cell-mediated immune response in BALB/c mice. In addition, they were recognized by sera from COVID-19-positive and/or vaccinated humans. Of note, the proteins of this study were designed based on the sequence of the Wuhan-Hu-1 isolate, and the human serum samples utilized in ELISA were collected between 2020 and 2021, when different variants were circulating in Brazil [56]. Our results reinforce the importance of continued research into protein construction techniques to improve the immunogenicity and efficacy of protein-based therapies and diagnostics.

## 4. Conclusions

The present study explored an approach based on the construction of recombinant proteins employing immunoinformatics and in silico structural analysis to identify conserved and immunogenic epitopes with broad recognition within the SARS-CoV-2 variants. Predicted T and B cell epitopes were selected to construct chimeric proteins containing immunogenic regions from the spike (S_epit) and nucleocapsid (N_epit) proteins of SARS-CoV-2. Both constructs elicited immune responses in mice and were recognized by sera from individuals who were COVID-19-positive and/or vaccinated. Thus, our study shows that S_epit and N_epit could be used for the development of novel immunological-based approaches to control SARS-CoV-2 infections.

## Data Availability

Data are contained within the article and Supplementary Materials.

## Supplementary Materials

The following supporting information can be downloaded at: https://www.mdpi.com/article/10.3390/biotech14020038/s1, Figure S1: Alignment of the amino acid sequence of the SARS-CoV-2 spike protein and its variants; Figure S2: Structural alignment between the predicted S_epit construct and the full-length SARS-CoV-2 spike protein, both generated using Modeller 10.6; Figure S3: Structural alignment between the predicted N_epit construct and the full-length SARS-CoV-2 nucleocapsid protein, both generated using Modeller 10.6; Figure S4: Western blot analysis of recombinant SARS-CoV-2 spike protein (S_epit) and nucleoprotein (N_epit) expressed in *Escherichia coli* BL21(DE) Star at 37 °C.

## References

[1] Zhou P., Yang X.L., Wang X.G., Hu B., Zhang L., Zhang W., Si H.R., Zhu Y., Li B., Huang C.L. (2020). A Pneumonia Outbreak Associated with a New Coronavirus of Probable Bat Origin. Nature.

[2] Lauxmann M.A., Santucci N.E., Autrán-Gómez A.M. (2020). The SARS-CoV-2 Coronavirus and the COVID-19 Outbreak. Int. Braz. J. Urol.

[3] Mingaleeva R.N., Nigmatulina N.A., Sharafetdinova L.M., Romozanova A.M., Gabdoulkhakova A.G., Filina Y.V., Shavaliyev R.F., Rizvanov A.A., Miftakhova R.R. (2022). Biology of the SARS-CoV-2 Coronavirus. Biochemistry.

[4] El-Maradny Y.A., Badawy M.A., Mohamed K.I., Ragab R.F., Moharm H.M., Abdallah N.A., Elgammal E.M., Rubio-Casillas A., Uversky V.N., Redwan E.M. (2024). Unraveling the Role of the Nucleocapsid Protein in SARS-CoV-2 Pathogenesis: From Viral Life Cycle to Vaccine Development. Int. J. Biol. Macromol..

[5] Alhamlan F.S., Al-Qahtani A.A. (2025). SARS-CoV-2 Variants: Genetic Insights, Epidemiological Tracking, and Implications for Vaccine Strategies. Int. J. Mol. Sci..

[6] Wang M.Y., Zhao R., Gao L.J., Gao X.F., Wang D.P., Cao J.M. (2020). SARS-CoV-2: Structure, Biology, and Structure-Based Therapeutics Development. Front. Cell. Infect. Microbiol..

[7] De Sanctis J.B., García A.H., Moreno D., Hajduch M. (2021). Coronavirus Infection: An Immunologists’ Perspective. Scand J. Immunol..

[8] Le Bert N., Tan A.T., Kunasegaran K., Tham C.Y.L., Hafezi M., Chia A., Chng M.H.Y., Lin M., Tan N., Linster M. (2020). SARS-CoV-2-Specific T Cell Immunity in Cases of COVID-19 and SARS, and Uninfected Controls. Nature.

[9] Weiskopf D., Immunol S., Weiskopf D., Schmitz K.S., Raadsen M.P., Grifoni A., Okba N.M.A., Van Den Akker J.P.C., Molenkamp R., Koopmans M.P.G. (2020). Phenotype and kinetics of SARS-CoV-2-specific T cells in COVID-19 patients with acute respiratory distress syndrome. Sci. Immunol..

[10] Li G., Fan Y., Lai Y., Han T., Li Z., Zhou P., Pan P., Wang W., Hu D., Liu X. (2020). Coronavirus Infections and Immune Responses. J. Med. Virol..

[11] Yadav R., Chaudhary J.K., Jain N., Chaudhary P.K., Khanra S., Dhamija P., Sharma A., Kumar A., Handu S. (2021). Role of Structural and Non-Structural Proteins and Therapeutic Targets of SARS-CoV-2 for COVID-19. Cells.

[12] Grifoni A., Sidney J., Zhang Y., Scheuermann R.H., Peters B., Sette A. (2020). A Sequence Homology and Bioinformatic Approach Can Predict Candidate Targets for Immune Responses to SARS-CoV-2. Cell Host Microbe.

[13] Vidal L.E.L., Figueira-Mansur J., Jurgilas P.B., Argondizzo A.P.C., Pestana C.P., Martins F.O., da Silva Junior H.C., Miguez M., Loureiro B.O., Marques C.d.F.S. (2023). Process Development and Characterization of Recombinant Nucleocapsid Protein for Its Application on COVID-19 Diagnosis. Protein Expr. Purif..

[14] Simões R.S.d.Q., Rodríguez-Lázaro D. (2022). Classical and Next-Generation Vaccine Platforms to SARS-CoV-2: Biotechnological Strategies and Genomic Variants. Int. J. Environ. Res. Public Health.

[15] Kovalenko A., Ryabchevskaya E., Evtushenko E., Nikitin N., Karpova O. (2023). Recombinant Protein Vaccines against Human Betacoronaviruses: Strategies, Approaches and Progress. Int. J. Mol. Sci..

[16] Tarke A., Grifoni A., Sette A. (2022). Bioinformatic and Experimental Analysis of T Cell Immune Reactivity to SARS-CoV-2 and Its Variants. Front. Bioinform..

[17] Deepthi V., Sasikumar A., Mohanakumar K.P., Rajamma U. (2025). Computationally Designed Multi-Epitope Vaccine Construct Targeting the SARS-CoV-2 Spike Protein Elicits Robust Immune Responses in Silico. Sci. Rep..

[18] Eswar N., John B., Mirkovic N., Fiser A., Ilyin V.A., Pieper U., Stuart A.C., Marti-Renom M.A., Madhusudhan M.S., Yerkovich B. (2003). Tools for Comparative Protein Structure Modeling and Analysis. Nucleic Acids Res..

[19] Šali A., Blundell T.L. (1993). Comparative Protein Modelling by Satisfaction of Spatial Restraints. J. Mol. Biol..

[20] Yuan M., Liu H., Wu N.C., Wilson I.A. (2021). Recognition of the SARS-CoV-2 Receptor Binding Domain by Neutralizing Antibodies. Biochem. Biophys. Res. Commun..

[21] Pettersen E.F., Goddard T.D., Huang C.C., Couch G.S., Greenblatt D.M., Meng E.C., Ferrin T.E. (2004). UCSF Chimera—A Visualization System for Exploratory Research and Analysis. J. Comput. Chem..

[22] Gasteiger E., Hoogland C., Gattiker A., Duvaud S., Wilkins M.R., Appel R.D., Bairoch A. (2005). Protein Identification and Analysis Tools on the ExPASy Server. The Proteomics Protocols Handbook.

[23] Doytchinova I.A., Flower D.R. (2007). VaxiJen: A Server for Prediction of Protective Antigens, Tumour Antigens and Subunit Vaccines. BMC Bioinform..

[24] Krogh A., Larsson B., Von Heijne G., Sonnhammer E.L.L. (2001). Predicting Transmembrane Protein Topology with a Hidden Markov Model: Application to Complete Genomes. J. Mol. Biol..

[25] Hall T. (1999). BioEdit: A User-Friendly Biological Sequence Alignment Editor and Analysis Program for Windows 95/98/NT. Nucleic Acids Symp. Ser..

[26] Green M.R., Sambrook J. (2021). Cloning and Transformation with Plasmid Vectors. Cold Spring Harb. Protoc..

[27] Laemmli U.K. (1970). Cleavage of Structural Proteins during the Assembly of the Head of Bacteriophage T4. Nature.

[28] Ghaemi A., Roshani Asl P., Zargaran H., Ahmadi D., Hashimi A.A., Abdolalipour E., Bathaeian S., Miri S.M. (2022). Recombinant COVID-19 Vaccine Based on Recombinant RBD/Nucleoprotein and Saponin Adjuvant Induces Long-Lasting Neutralizing Antibodies and Cellular Immunity. Front. Immunol..

[29] Nazarian S., Olad G., Abdolhamidi R., Motamedi M.J., Kazemi R., Kordbacheh E., Felagari A., Olad H., Ahmadi A., Bahiraee A. (2022). Preclinical Study of Formulated Recombinant Nucleocapsid Protein, the Receptor Binding Domain of the Spike Protein, and Truncated Spike (S1) Protein as Vaccine Candidates against COVID-19 in Animal Models. Mol. Immunol..

[30] Chen W.H., Pollet J., Strych U., Lee J., Liu Z., Kundu R.T., Versteeg L., Villar M.J., Adhikari R., Wei J. (2022). Yeast-Expressed Recombinant SARS-CoV-2 Receptor Binding Domain RBD203-N1 as a COVID-19 Protein Vaccine Candidate. Protein Expr. Purif..

[31] Rabdano S.O., Ruzanova E.A., Pletyukhina I.V., Saveliev N.S., Kryshen K.L., Katelnikova A.E., Beltyukov P.P., Fakhretdinova L.N., Safi A.S., Rudakov G.O. (2023). Immunogenicity and In Vivo Protective Effects of Recombinant Nucleocapsid-Based SARS-CoV-2 Vaccine Convacell^®^. Vaccines.

[32] Primard C., Monchâtre-Leroy E., Del Campo J., Valsesia S., Nikly E., Chevandier M., Boué F., Servat A., Wasniewski M., Picard-Meyer E. (2023). OVX033, a Nucleocapsid-Based Vaccine Candidate, Provides Broad-Spectrum Protection against SARS-CoV-2 Variants in a Hamster Challenge Model. Front. Immunol..

[33] Silva E.K.V.B., Bomfim C.G., Barbosa A.P., Noda P., Noronha I.L., Fernandes B.H.V., Machado R.R.G., Durigon E.L., Catanozi S., Rodrigues L.G. (2022). Immunization with SARS-CoV-2 Nucleocapsid Protein Triggers a Pulmonary Immune Response in Rats. PLoS ONE.

[34] Wu A., Peng Y., Huang B., Ding X., Wang X., Niu P., Meng J., Zhu Z., Zhang Z., Wang J. (2020). Genome Composition and Divergence of the Novel Coronavirus (2019-NCoV) Originating in China. Cell Host Microbe.

[35] Nithya Shree J., Premika T., Sharlin S., Annie Aglin A. (2024). Diverse Approaches to Express Recombinant Spike Protein: A Comprehensive Review. Protein Expr. Purif..

[36] Ghaderi H., Shoari A., Salehi S., Eskafi A.H., Habibi-Anbouhi M., Cohan R.A., Moazzami R., Behdani M. (2024). Expression and Purification of SARS-CoV-2 Receptor Binding Domain in Escherichia Coli for Diagnostic and Therapeutic Purposes. Res. Pharm. Sci..

[37] Djukic T., Mladenovic M., Stanic-Vucinic D., Radosavljevic J., Smiljanic K., Sabljic L., Devic M., Cujic D., Vasovic T., Simovic A. (2021). Expression, Purification and Immunological Characterization of Recombinant Nucleocapsid Protein Fragment from SARS-CoV-2. Virology.

[38] Bhatwa A., Wang W., Hassan Y.I., Abraham N., Li X.Z., Zhou T. (2021). Challenges Associated with the Formation of Recombinant Protein Inclusion Bodies in Escherichia Coli and Strategies to Address Them for Industrial Applications. Front. Bioeng. Biotechnol..

[39] Chan P., Curtis R.A., Warwicker J. (2013). Soluble Expression of Proteins Correlates with a Lack of Positively-Charged Surface. Sci. Rep..

[40] Kamtekar S., Schiffer J.M., Xiong H., Babik J.M., Hecht M.H. (1993). Protein Design by Binary Patterning of Polar and Nonpolar Amino Acids. Science.

[41] Falak S., Sajed M., Rashid N. (2022). Strategies to Enhance Soluble Production of Heterologous Proteins in Escherichia Coli. Biologia.

[42] Qing R., Hao S., Smorodina E., Jin D., Zalevsky A., Zhang S. (2022). Protein Design: From the Aspect of Water Solubility and Stability. Chem. Rev..

[43] Wang C., Zou Q. (2023). Prediction of Protein Solubility Based on Sequence Physicochemical Patterns and Distributed Representation Information with DeepSoluE. BMC Biol..

[44] Zhang Z.X., Nong F.T., Wang Y.Z., Yan C.X., Gu Y., Song P., Sun X.M. (2022). Strategies for Efficient Production of Recombinant Proteins in Escherichia Coli: Alleviating the Host Burden and Enhancing Protein Activity. Microb. Cell Fact..

[45] Ramos F.F., Pereira I.A.G., Cardoso M.M., Bandeira R.S., Lage D.P., Scussel R., Anastacio R.S., Freire V.G., Melo M.F.N., Oliveira-da-Silva J.A. (2023). B-Cell Epitopes-Based Chimeric Protein from SARS-CoV-2 N and S Proteins Is Recognized by Specific Antibodies in Serum and Urine Samples from Patients. Viruses.

[46] Silva M.d.O., Castro-Amarante M.F., Venceslau-Carvalho A.A., Almeida B.d.S., Daher I.P., Souza-Silva G.A.d., Yamamoto M.M., Koike G., de Souza E.E., Wrenger C. (2024). Enhanced Immunogenicity and Protective Effects against SARS-CoV-2 Following Immunization with a Recombinant RBD-IgG Chimeric Protein. Vaccines.

[47] D’Atri V., Imiołek M., Quinn C., Finny A., Lauber M., Fekete S., Guillarme D. (2024). Size Exclusion Chromatography of Biopharmaceutical Products: From Current Practices for Proteins to Emerging Trends for Viral Vectors, Nucleic Acids and Lipid Nanoparticles. J. Chromatogr. A.

[48] Feng W., Xiang Y., Wu L., Chen Z., Li Q., Chen J., Guo Y., Xia D., Chen N., Zhang L. (2022). Nucleocapsid Protein of SARS-CoV-2 Is a Potential Target for Developing New Generation of Vaccine. J. Clin. Lab. Anal..

[49] Rak A., Isakova-Sivak I., Rudenko L. (2023). Overview of Nucleocapsid-Targeting Vaccines against COVID-19. Vaccines.

[50] Dobaño C., Santano R., Jiménez A., Vidal M., Chi J., Rodrigo Melero N., Popovic M., López-Aladid R., Fernández-Barat L., Tortajada M. (2021). Immunogenicity and Crossreactivity of Antibodies to the Nucleocapsid Protein of SARS-CoV-2: Utility and Limitations in Seroprevalence and Immunity Studies. Transl. Res..

[51] De Marco Verissimo C., O’Brien C., López Corrales J., Dorey A., Cwiklinski K., Lalor R., Doyle J.M., Field S., Masterson C., Martinez E.R. (2021). Improved Diagnosis of SARS-CoV-2 by Using Nucleoprotein and Spike Protein Fragment 2 in Quantitative Dual ELISA Tests. Epidemiol. Infect..

[52] Zhang L., Zheng B., Gao X., Zhang L., Pan H., Qiao Y., Suo G., Zhu F. (2020). Development of Patient-Derived Human Monoclonal Antibodies Against Nucleocapsid Protein of Severe Acute Respiratory Syndrome Coronavirus 2 for Coronavirus Disease 2019 Diagnosis. Front. Immunol..

[53] Schunk M.K., Macallum G.E. (2005). Applications and Optimization of Immunization Procedures. ILAR J.

[54] Mamat U., Wilke K., Bramhill D., Schromm A.B., Lindner B., Kohl T.A., Corchero J.L., Villaverde A., Schaffer L., Head S.R. (2015). Detoxifying Escherichia Coli for Endotoxin-Free Production of Recombinant Proteins. Microb. Cell Fact..

[55] Shahar E., Emquies K., Bloch I., Eliahu D., Ben Adiva R., Pitcovski J., Yadid I. (2023). Endotoxin-Free Gram-Negative Bacterium as a System for Production and Secretion of Recombinant Proteins. Appl. Microbiol. Biotechnol..

[56] Alcantara L.C.J., Nogueira E., Shuab G., Tosta S., Fristch H., Pimentel V., Souza-Neto J.A., Coutinho L.L., Fukumasu H., Sampaio S.C. (2022). SARS-CoV-2 Epidemic in Brazil: How the Displacement of Variants Has Driven Distinct Epidemic Waves. Virus Res..

