# Supplementary material for: Strategy for the Construction of SARS-CoV-2 S and N Recombinant Proteins and Their Immunogenicity Evaluation
Source: BioTech (Basel). 2025 May 23;14(2):38. doi: 10.3390/biotech14020038 (PMC12191430; doi:10.3390/biotech14020038)
Supplement: Supplementary file 1 [file biotech-14-00038-s001.zip › biotech-3485589-supplementary.pdf]

BioTech

Article

Strategy for the Construction of SARS-CoV-2 S and N Recombinant Proteins and Their Immunogenicity Evaluation

Paulo Henrique Guilherme Borges [1,†], Barbara Gregio [1,†], Helena Tiemi Suzukawa [2], Gislaine Silva-Rodrigues [1], Emanuella de Castro Andreassa [3], Isabela Madeira de Castro [1], Guilherme Bartolomeu-Gonçalves [4], Emerson José Venancio [5], Phileno Pinge-Filho [5], Viviane Monteiro Góes [6], Celso Vataru Nakamura [7], Eliandro Reis Tavares [2,8], Tatiana de Arruda Campos Brasil de Souza [3], Sueli Fumie Yamada-Ogatta [2] and Lucy Megumi Yamauchi [2,*]

Supplementary Materials:

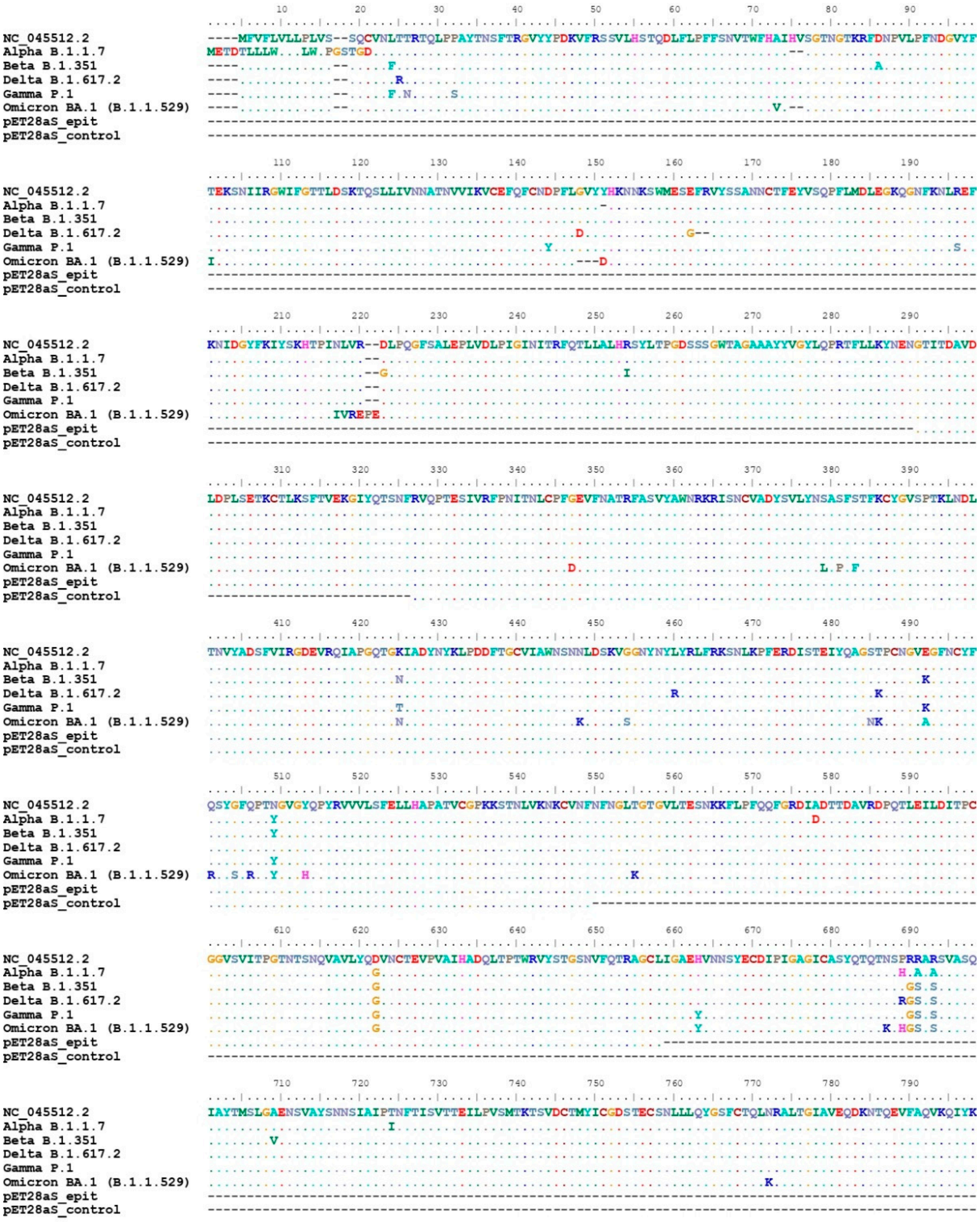

 https://doi.org/10.3390/biotech14020038

Figure S1. Alignment of the amino acid sequence of the SARS-CoV-2 spike protein and its variants. The alignment was performed using the amino acid sequence of the SARS-CoV-2 spike protein isolated in Wuhan (NC_045512.2), the alpha (B.1.1.7), beta (B.1.351), delta (B.1.617.2), gamma (P.1) and omicron (BA.1) variants, and the two sequences used to construct the vectors the pET28aS_epit and pET28aS_control vectors.

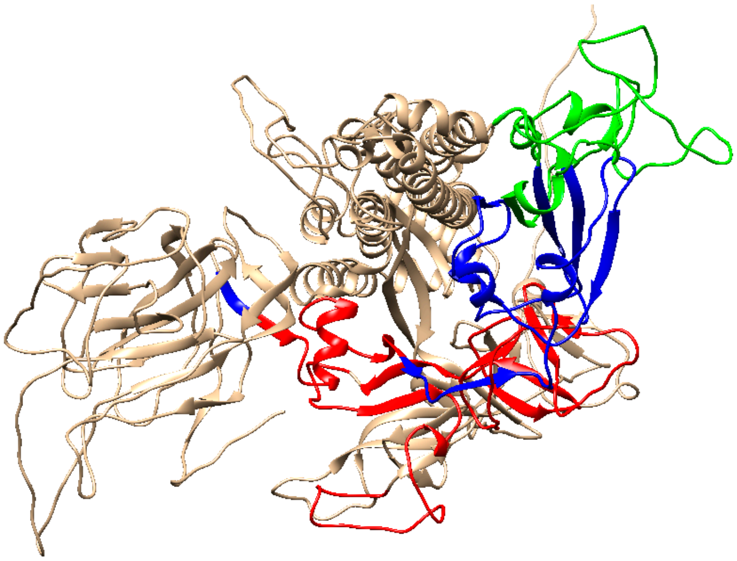

Figure S2. Structural alignment between the predicted S_epit construct and the full-length SARS-CoV-2 spike protein, both generated using Modeller 10.6 [19]. The full protein structure is rendered in wheat. The selected immunogenic epitopes are highlighted as follows: residues 287–317 (red), 402–507 (green), 524–598 (red), and 601–640 (red). Structural elements outside epitope regions included for stability are shown in blue.

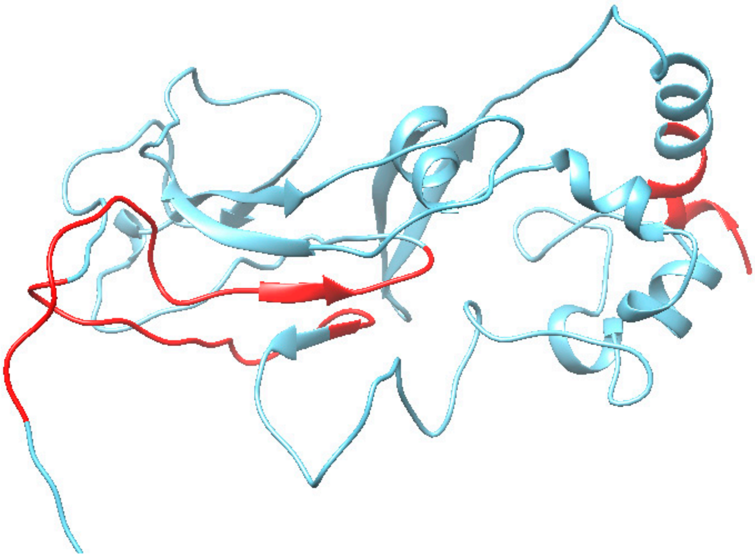

Figure S3. Structural alignment between the predicted N_epit construct and the full-length SARS-CoV-2 nucleocapsid protein, both generated using Modeller 10.6 [19]. The three selected epitopes (residues 42–62, 153–172, and 355–401) are shown in red and are linked by flexible glycine loops. The full-length nucleoprotein in light blue.

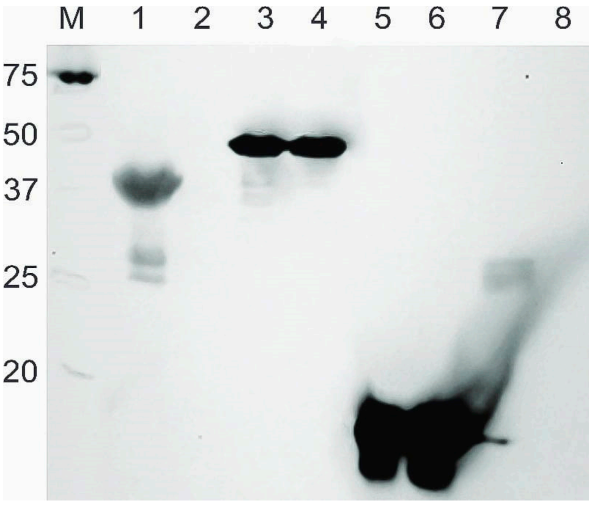

Figure S4. Western blot analysis of recombinant SARS-CoV-2 spike protein (S_epit) and nucleoprotein (N_epit) expressed in *Escherichia coli* BL21 (DE3) star at 37 °C. The proteins were analyzed using monoclonal anti-histidine antibodies as primary antibodies, and alkaline phosphatase-conjugated anti-mouse antibodies as secondary antibodies. Molecular weight marker (M, BioRad, 250 kDa); the total (1) and soluble (2) protein extracts of the S_epit (39 kDa); the total (3) and soluble (4) extracts of the N_control (47 kDa), the total (5) and soluble (6) extracts of the N_epit (15 kDa), and the total (7) and soluble (8) extracts of the S_control (25 kDa).
